# SA Rugby Injury and Illness Surveillance and Prevention Project (SARIISPP): *The Currie Cup Premiership Competition Injury Surveillance Report* 2019

**DOI:** 10.17159/2078-516X/2020/v32i1a8560

**Published:** 2020-01-01

**Figure:**
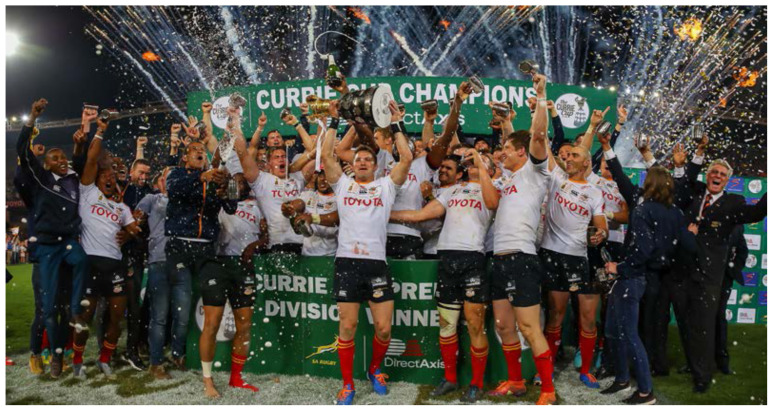


## Executive Summary

As part of the SA Rugby Injury and Illness Surveillance and Prevention Project (SARIISPP), The Currie Cup 2019 Premiership Division Competition (‘The Currie Cup’) injury data were recorded throughout the tournament by the medical doctors and medical support staff of the respective teams. All seven teams were required to record every match injury that occurred in their team. Only match injuries and match time exposure for each participating team were considered for this report.

The injury rate of Time-Loss injuries for The Currie Cup 2019 was 94 (74 to 113) injuries per 1000 player hours (mean and 95% confidence intervals – see p8 for explanation), which is higher than the international rate of 81 (63 to 105) injuries per 1000 player hours[1], and equates to 1.9 injuries per team per match.

The Toyota Free State Cheetahs had the highest injury rate for Time-Loss injuries for the 2019 tournament, but this was not significantly higher than any other team, or the 2019 tournament average. The Phakisa Pumas had the lowest injury rate for Time-Loss injuries for the 2019 tournament, with their 2019 injury rate being significantly lower than their 2015–2018 tournament average. This finding is interesting to note as the 2019 tournament was won by the Toyota Free State Cheetahs, with the Phakisa Pumas ranking at the bottom of the log at the end of the tournament. In previous years the teams who ranked in 1^st^ or 2^nd^ positions of the competition had significantly lower injury rates than those who ranked in last position[2]. As such the injury rates in the 2019 tournament reflected an opposite trend to previous years. While the Toyota Free State Cheetahs had the highest number of Time-Loss injuries in 2019 they were second lowest on *average* severity and the Phakisa Pumas, with the lowest number of Time-Loss injuries, had the highest *average* severity. What this means is that although the Toyota Free State Cheetahs had the most injuries, they did not lose many days of training and match play due to injury, while the Phakisa Pumas would have lost several player training and match days due to their injuries. Although teams may have a low injury rate, injuries of a high severity still represent a sizable burden to the team, resulting in many training and match days lost due to injury for that team. This highlights the importance of collecting severity data, and not injury rates on their own.

During The Currie Cup 2019 tournament, injuries estimated at *1–7 days* severity occurred at a higher rate than for the 2014–2018 tournaments, although this was not significant. Comparison of estimated vs actual severity of injuries for The Currie Cup 2019 showed that doctors and medical support staff slightly overestimated the number of injuries of *1–7 days* severity. The average severity of Time-Loss injuries in the 2019 tournament was 13 days, which is lower than the 37 days reported in the England Professional Rugby Injury Surveillance Project 2018 report[3]. The median injury severity of all Time-Loss injuries was 4 days with 25% of injuries losing 3 days or less and 25% of injuries losing 10 days or more due to injury.

For the first time contusion/bruise injuries were the most common injury type, with muscle (rupture/strain/tear) and sprained ligament injuries recording the second and third most common injury types, respectively. The head remained the most commonly injured body location. Concussion was the most common injury diagnosis for the fourth consecutive year. The tackle remained the most common injury event for the sixth consecutive year, with the incidence of tackle-related injuries similar to the 2014–2018 average.

In February 2020, the International Olympic Committee (IOC) published an updated consensus statement for methods of recording and reporting epidemiological data on injury and illness in sport[4]. The Currie Cup 2019 report has aligned with this more recent consensus statement. In certain sections of this report, such as the subsequent injury section for example, additional details on injuries have been added to assist practitioners when they report these injuries. Details on these additions are described in the Definitions section on p8.

**Figure:**
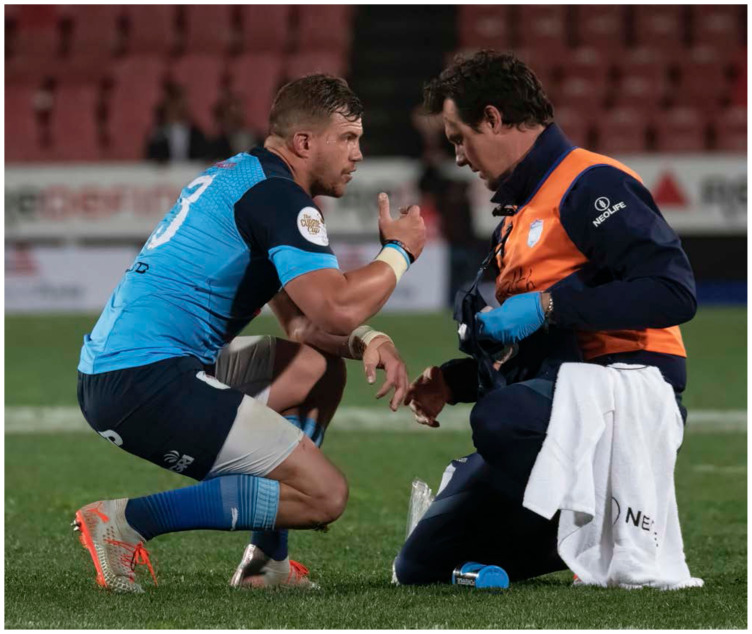


## Introduction

In 2014, as part of the SA Rugby Injury and Illness Surveillance and Prevention Project (SARIISPP), the South African Rugby Union (SA Rugby) implemented a new standardised injury surveillance format for The Currie Cup Premiership Division Competition. This required the team doctor or medical support staff to electronically capture all relevant match injury data on an Application (App) on their cell-phone or tablet. The App provided doctors or medical support staff with the standardised *BokSmart* injury surveillance data capture format, which is aligned with the IOC consensus statement for injury recording in sport [4] and for rugby union [5].

Injury surveillance is the critical first step in the development of injury prevention strategies. Injury surveillance captured in the correct format enables the comparison of injury rates between teams within the same tournament, tournament injuries over successive years, and with other rugby injury surveillance studies. Literature describing tournament injuries presents the injury numbers as a rate where the total number of injuries is divided by the total amount of time exposed to the risk of experiencing an injury. An injury rate is expressed as the number of events per 1000 player hours. This normalised version of the injury number facilitates comparison between The Currie Cup teams in 2019, previous tournaments and the international injury surveillance literature. Throughout this report the normalised injury rates have been provided to allow for comparison with other tournaments and the international literature, as discussed, but every effort has been made to present these rates on a ‘per team’ and ‘per match’ level for easier and more pragmatic interpretation.

Since 2016, The Currie Cup doctors or medical support staff were asked to record the physical return to play date of the injured players, thereby allowing for the actual severity of the injury to be calculated. For those cases, where the player had not returned to play by the start of the following year, doctors or medical support staff were asked to provide an estimated return to play date. The severity of these injuries was then calculated using the estimated date provided, and not the actual date. Calculating the actual severity of most injuries adds substantial value to the report as it enables one to determine the burden of the teams’ injuries with greater accuracy. Injury burden is a combination of the injury rate and severity and is expressed as the number of days’ absent from training and matches per 1000 player hours.

It is important to note that a multitude of factors contribute to players’ injury risk and injury causing events. The medical, conditioning, coaching staff and the players themselves are equally responsible for ensuring that players are medically, mentally and physically fit to handle the demands of the competition. Additionally, each player has unique intrinsic and extrinsic injury risk factors, which are beyond the control of the team’s staff.

An inherent issue with most injury surveillance studies is that the teams’ medical doctors or medical support staff are exclusively responsible for entering their team’s injury data. As no audit process is done on the collection of this data, in many of these cases, the accuracy of the data is dependent on the compliance of the doctors or medical support staff. This potential limitation is present in most injury surveillance studies. To minimise this potential limitation, SARIISPP had a project coordinator who was in frequent contact with the doctors or medical support staff to ensure they were up to date with the data capturing.

In 2019, The Currie Cup semi-finals were contested between Toyota Free State Cheetahs vs Cell C Sharks and Tafel Lager Griquas vs. Xerox Golden Lions. The final was between Toyota Free State Cheetahs vs Xerox Golden Lions, with the Toyota Free State Cheetahs eventually winning the tournament.

## Definitions

All definitions are originally based on the 2007 consensus statement for injury reporting in rugby union[5] and have since been realigned with the latest International Olympic Committee (IOC) consensus statement for methods of recording and reporting epidemiological data on injury and illness in sport[4].

### MEDICAL ATTENTION INJURY

All injuries that were seen by the teams’ doctor or medical support staff were classified as Medical Attention injuries, which are defined by the 2007 statement as an “*injury that results in a player receiving medical attention”* [5], and by the more recent IOC statement as *“a health problem that results in an athlete receiving medical attention”* [4].

### TIME-LOSS INJURY

Medical Attention injuries were further categorised as Time-Loss injuries, where appropriate, and defined by the 2007 statement as, “*an injury that results in a player being unable to take a full part in future rugby training or match play*” [5]. The IOC definition is, *“a health problem that results in a player being unable to complete the current or future training session or competition”* [4].

### INJURY RATE

For this report, an injury rate is the number of injuries expressed per 1000 player exposure hours. This method of expressing injury rate has been used in previous years’ reports of The Currie Cup Premiership tournament and other international literature, and therefore makes comparisons easy. Moreover, the injury rate is expressed as a mean with 95% confidence intervals. A 95% confidence interval around a mean value indicates that there is a 95% chance (i.e. very high chance) that the true value falls within this range. In this report, we present the 95% confidence intervals assuming normal distribution of the data and use the approach of examining the overlap of the confidence intervals to determine whether the injury incidences are significantly different; if the range of confidence interval values of two comparisons do not overlap, there is a strong chance (95%) that their injury rates are different from each other. We have opted for this method because it is easy to use, conservative and less likely to produce false positive results[6].

### MEDIAN (INTERQUARTILE RANGE)

When numbers are ordered from the lowest to highest, the median is the value which separates the higher half of the values from the lower half of the values. Simply put, it is the middle value of a list of ranked numbers. The interquartile range (IQR) describes the spread of the data. When rank ordered data are divided into quartiles the first and the third quartile represents the value under which 25% and 75% of the data points fall respectively. As an example, a team may have a median injury severity of 32 days (IQR 7 to 40). This means that when the teams’ injury severities are rank ordered the mid-point or median of the injury severities is 32 days. Also 25% of their injuries result in 7 or less days absent from training and matches and 25% of their injuries result in 40 days or more absent from training and matches.

### NEW, SUBSEQUENT AND RECURRENT INJURIES

In 2019, in The Currie Cup Premiership Division Competition, a ‘*New Injury’* was defined as when a player sustained his first injury. Any injury that the *same* player sustained after this initial injury was defined as a *‘Subsequent Injury’*.

According to the more recent IOC statement, any subsequent injury to the same site and of the same type is referred to as a ‘*Recurrence’* if the index injury was fully recovered before reinjury, and as an *‘Exacerbation’* if the index injury was not yet fully recovered[4].

To provide more detail on the subsequent injuries for practitioners, we have further categorized the subsequent injuries in this report into one of four groups based on the OSICS classification diagnosis:

- Different site - Different type
- Different site - Same type
- Same site - Different type
- Same site - Same type

According to the 2007 Consensus Statement for rugby, any subsequent injury classified as ‘Same site - Same type’ was a *‘Recurrent injury’* [5].

### INJURY SEVERITY

The total severity of an injury is defined as *“the number of days that have elapsed from the date of injury to the date of the player’s return to full participation in team training and availability for match selection”* [4], [5]. For each year, at the time of injury the doctors or medical support staff were asked to estimate the severity of the injury based on their clinical assessment of the injured player. These estimations were made according to the severity groupings provided in the 2007 consensus statement; *Slight* (0–1 days lost), *Minimal* (2–3 days lost), *Mild* (4–7 days lost), *Moderate* (8–28 days lost), *Severe* (>28 days lost), *Career ending* and *Non-fatal catastrophic* [5]. To align with the latest IOC statement the injuries have been re-grouped to reflect the severity groupings *‘1–7 days’, ‘8–28 days’ and ‘>28 days’*[4].

The average severity represents the average number of days lost per injury when dividing the accumulated total number of days lost by the total number of injury events. For example, a team may have a total severity of 550 days absent, accumulated from 22 injuries. The average severity of the team’s injuries would therefore be 550/22, which equals, on average 25 days absent per injury.

### INJURY BURDEN

Injury burden is a combination of injury rate and severity. It is the injury rate multiplied by the average severity (number of days lost due to injury) and is expressed as the number of days absent per 1000 player hours. For example, a team who has an injury rate of 75 injuries per 1000 player exposure hours, and an average severity of 38 days lost per injury will have an injury burden of 2850 days absent per 1000 player hours (75 × 38).

### OPERATIONAL INJURY BURDEN

The operational burden is the expected number of days lost per injury per team for every match played over the tournament or season. The measure is an extrapolation of injury rates and severities over a season and includes the most severe injuries together with the least severe injuries in its estimation. For example, if a team has an operational injury burden of 2 days, it means that based on their injury rates and average severity, on average, 2 days absence can be expected from every match injury the team sustains.

### META-ANALYSIS

A meta-analysis is a study using statistical methods to combine multiple scientific studies with varying levels of evidence on the same topic to determine overall defining patterns and results from the combined data. As such, it represents the highest level of scientific evidence available. The findings in this report are compared to that of the most recent meta-analysis for rugby union injuries at a senior professional level[1]. Although this was published in 2013, it remains the most comprehensive assessment of injuries associated with rugby.

**Figure:**
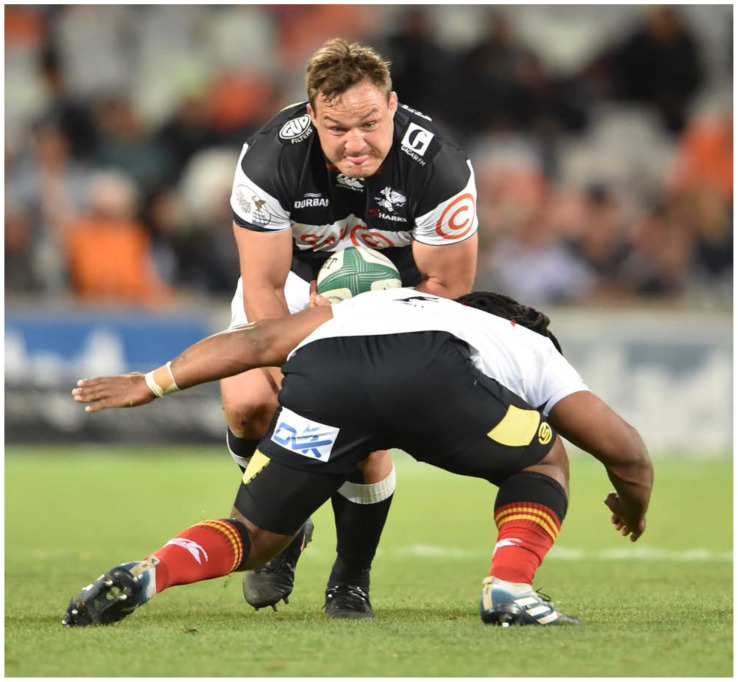


## Key Findings

### Injured players

During ‘The Currie Cup’ 2019, 75 players sustained a total of 90 Time-Loss injuries. This means that of the 161 players exposed to playing rugby in the tournament (7 teams × 23 players per match-day squad), 47% sustained an injury at some stage in the tournament (Figure 1a). The proportion of players who experienced one Time-Loss injury decreased from 2018 to 2019, while the proportion of players who experienced 2 or 3 injuries increased from 2018 to 2019 (Figure 1b). Further analyses will focus on absolute injury numbers, regardless of the number of players who sustained them.

### Overall injury rate

Only the number of Time-Loss injuries, those which resulted in the player missing more than one training session or match, were considered for further analysis, because these injuries are more comparable between different teams, tournaments and with the published scientific literature [5].

Overall, the match injury rate for all Time-Loss injuries in 2019 for The Currie Cup was 94 (74 to 113) injuries per 1000 player exposure hours. This is higher than the rate of the meta-analysis (81 injuries per 1000 player hours, 63 to 105) [1]. An injury rate of 94 injuries per 1000 player hours equates to 1.9 injuries per team per match.

When ranking the teams from highest to lowest injury rate for Time-Loss injuries in 2019, the Toyota Free State Cheetahs had the highest injury rate and the Phakisa Pumas had the lowest injury rate. The Time-Loss injury rate of the Toyota Free State Cheetahs was significantly higher than the Phakisa Pumas, but not significantly higher than the tournament average (Figure 2a).

The average severity of match injuries for The Currie Cup 2019 was 13 days, which is lower than the average severity for match injuries of 37 days reported in the England Professional Rugby Injury Surveillance Project 2017 – 2018 season report[3]. The median severity of all Time-Loss injuries was 4 days (IQR 3 to 10). This means that the half-way mark of the injury severities was 4 days, with 25% of all Time-Loss injuries lasting for 3 days or less and 25% lasting 10 days or longer.

Table 1 displays the injury rates of the Time-Loss injuries for each team (shown in Figure 2a), as the number of incidents per match and per player. The Vodacom Blue Bulls have been used as a worked example to explain the Table. The Vodacom Blue Bulls had an injury rate of 92 injuries per 1000 player hours, which equates to 1.8 injuries per match. Another way to describe this is that for every 10.9 player match hours the Vodacom Blue Bulls sustained one match injury. At the start of each match if all players had an equal chance of injury, each player in the Vodacom Blue Bulls team would have a 12% chance of injury for each match played. With a 12% chance of injury at each match, a player in the Vodacom Blue Bulls team can expect to be injured once in every 8.2 matches played (Table 1). The information in this Table will be unfolded in the subsequent graphs.

When comparing the team’s 2014–2018 averaged tournament injury rate to their 2019 injury rate, the Phakisa Pumas experienced a significantly lower injury rate in 2019 in comparison to their 2015–2018 tournament average (Figure 2b; they never played in this division in 2014). The Toyota Free State Cheetahs and Xerox Golden Lions were notably higher than the previous years’ averaged rates, but these were not significantly different.

It remains interesting to note that the combined mean and 95% CI for all teams for all years, 83 (77 to 89) injuries per 1000 player hours is similar to the summary of international data described in the meta-analysis of 81 (63 to 105) injuries per 1000 player hours[1].

The data in this report is aligned with the most recent IOC consensus statement[4] and is further presented such that it facilitates comparison with previous season reports and the meta-analysis[1]. Tables 2 and 3 present The Currie Cup 2019 injury data in the format recommended by the most recent IOC consensus statement. This provides an overview of the 2019 season’s data in this format, with the data explored in more detail throughout the report.

### Average Time-Loss Injury rates per Month of the Tournament

**Figure 3:**
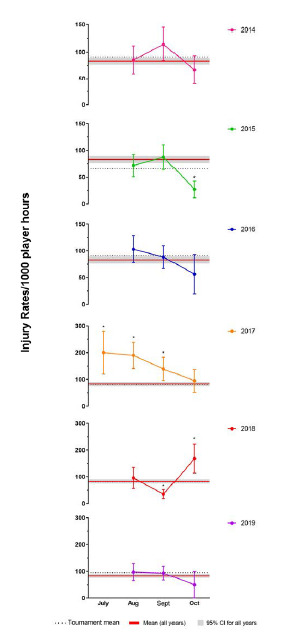
The Time–Loss Injury rates per month for each of The Currie Cup 2014 – 2019 tournaments with mean and 95% confidence intervals (CI) displayed for all teams and for all years combined (red line). Asterisk (*) indicates injury rates are significantly different to the mean tournament average.

In 2019, there was a similar trend compared to previous years (except 2018), with the lowest injury rate being recorded in the last month of the tournament. In The Currie Cup 2019, no month’s injury rate was significantly different from the rest (Figure 3).

### New, Subsequent and Recurrent Injuries

Overall, the injury rate for New Time-Loss injuries for The Currie Cup 2019 was 78 (60 to 96) injuries per 1000 player hours, which is similar to that of the meta-analysis[1] with a rate of 78 (74 to 83) injuries per 1000 player hours. The average severity for New Time-Loss Injuries in The Currie Cup 2019 was 15 (9 to 21) days, which is lower than the average severity reported in the meta-analysis[1] of 20 (15 to 24) days.

There were 13 players who sustained more than one injury in The Currie Cup 2019. The majority (n = 10, 67%) of the subsequent Time-Loss injuries were at a different anatomical site and of a different type when compared to the first injury (Figure 4a & 4b).

A subsequent recurrent injury was any subsequent injury classified as ‘same site-same type’, which refers to the same location and same tissue type involved as the original index injury. There were only two subsequent recurrent injuries in The Currie Cup 2019. The overall injury rate for subsequent recurrent Time-Loss injuries was 2 (1 to 5) injuries per 1000 player hours, which is significantly lower than the meta-analysis rate of 11 (10 to 12) injuries per 1000 player hours[1]. The severity of the two recurrent injuries in 2019 was 3 and 4 days lost respectively.

**Figure:**
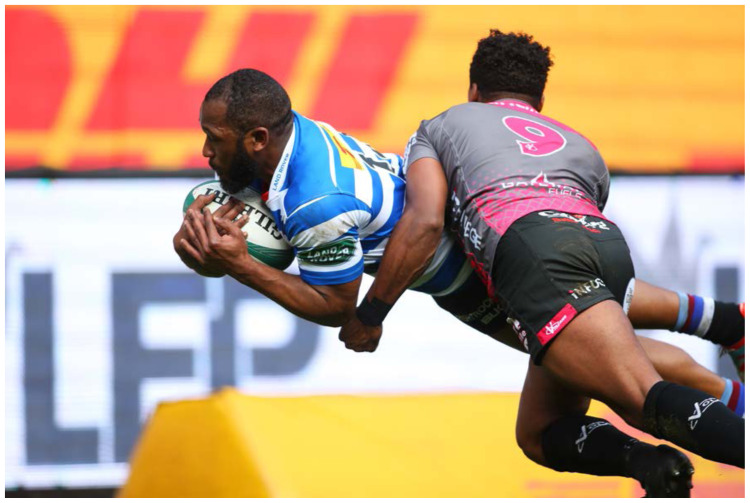


**Figure 5:**
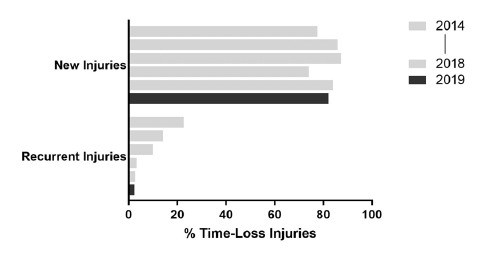
Proportion of new versus subsequent recurrent injuries for The Currie Cup 2014 – 2019 tournaments. Data expressed as a % of Time-Loss injuries for 2014 (n = 120), 2015 (n = 114), 2016 (n = 142), 2017 (n = 126), 2018 (n = 77) and 2019 (n = 90).

When comparing the new and subsequent recurrent injuries across The Currie Cup 2014 – 2019 tournaments, there was a decrease in the proportion of subsequent recurrent injuries over the five years (Figure 5). This finding must be interpreted with caution as in 2014 and 2015 the classification of a subsequent recurrent injury was made by the doctor or medical support staff; thus, the definition of a subsequent recurrent injury was open to interpretation by the doctor or medical support staff. From 2016 onwards however, an injury was classified as a subsequent recurrent injury based on the OSICS classification diagnosis[7].

**Figure:**
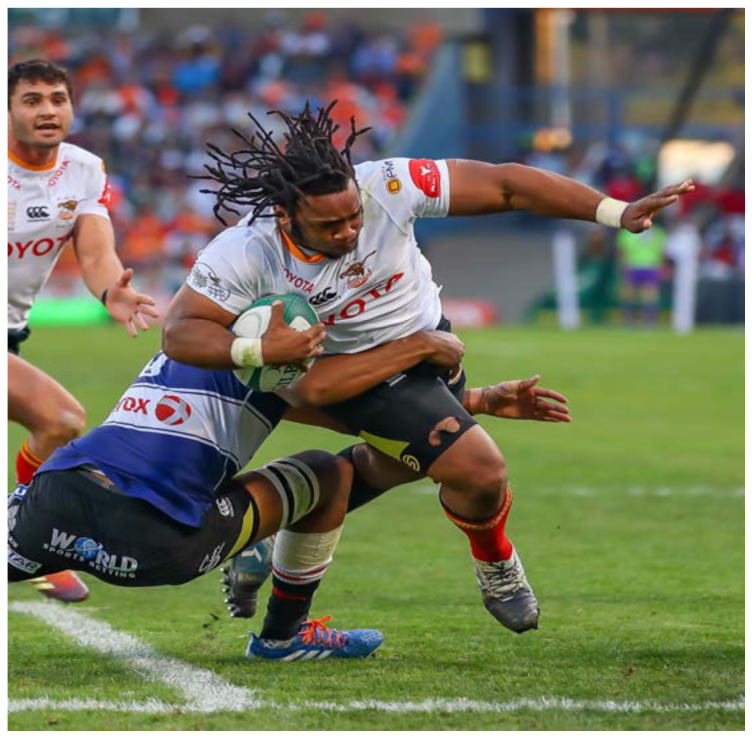


### Injury Severity

For each year, at the time of injury the doctors or medical support staff were asked to estimate the severity of the injury based on their clinical assessment of the injured player. A *‘Slight’* injury refers to 0–1 days, *‘Minimal’* is 2–3 days, *‘Mild’* is 4–7 days, *‘Moderate’* is 8–28 days and *‘Severe’* is >28 days off rugby training and/or match play. These severity groupings are according to the original 2007 consensus statement for rugby union[5]. To align with the latest IOC statement, the injuries have been re-grouped to reflect the severity groupings *‘1–7 days’, ‘8–28 days’ and ‘>28 days’*[4].

**Figure 6:**
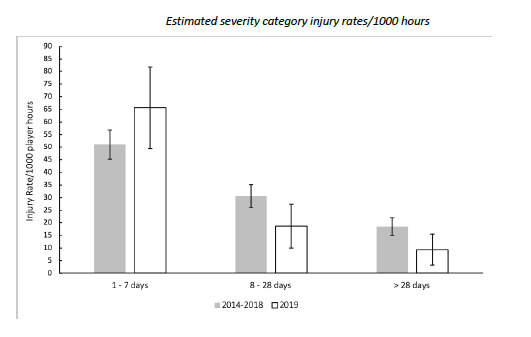
The estimated severity category injury rates of Time-Loss injuries for The Currie Cup 2019 in comparison to the averaged injury rates for the 2014–2018 estimated severity categories. Estimated severity category injury rates/1000 hours

Figure 6 compares the estimated injury severity rates for The Currie Cup 2019 tournament to the 2014–2018 tournament averaged rate. There were no significantly different injury rates in 2019 in comparison to previous years (Figure 6).

**Figure 7:**
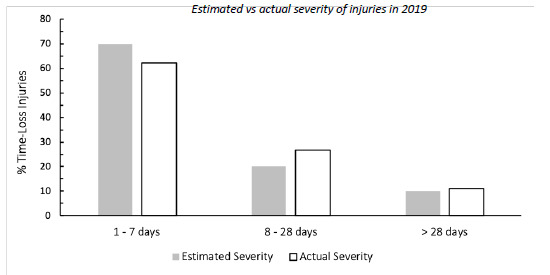
The estimated and actual severity of Time-Loss injuries for The Currie Cup 2019. Data expressed as a proportion of all Time-Loss Injuries (n = 90).

Since The Currie Cup 2016, doctors or medical support staff were asked to record the physical return to play dates of their injured players, which allowed for the actual injury severity to be calculated. Figure 7 compares the estimated severity recorded by the doctor or medical support staff at the time of injury and the actual injury severity determined once the player had returned from injury. In The Currie Cup 2019, medical staff slightly over-estimated the number of *‘1–7 days’* injuries and under-estimated the number of ‘*8–28 days’* injuries. Medical staff estimated 20% of Time-Loss injuries to be ‘*8–28 days’* while 26% were of ‘*8–28 days’* severity.

Table 4 describes the actual severity of each teams’ Time-Loss injuries for The Currie Cup 2019. The Vodacom Blue Bulls have again been used as a worked example to explain the Table. The Vodacom Blue Bulls sustained 1.8 injuries per match, meaning that for every 0.5 matches played they sustained one injury. In total, the Vodacom Blue Bulls lost 67 training and match days due to injury. This equates to an average of 6 training and match days lost for every injury sustained. The burden of the team’s injuries equates to 560 days lost per 1000 player hours. Translating this to an operational burden per match, it shows that the Vodacom Blue Bulls lost 11 days per injury per match over the season. The median injury severity for the Vodacom Blue Bulls was 9 days (IQR 2 to 9). This means that when severities of the Vodacom Blue Bulls Time-Loss injuries were rank ordered the midpoint of the severities was 9 days off from rugby, with 25% of their injuries lasting equal to or less than 2 days off and 25% of their injuries lasting equal to or longer than 9 days off (Table 4).

**Figure 8:**
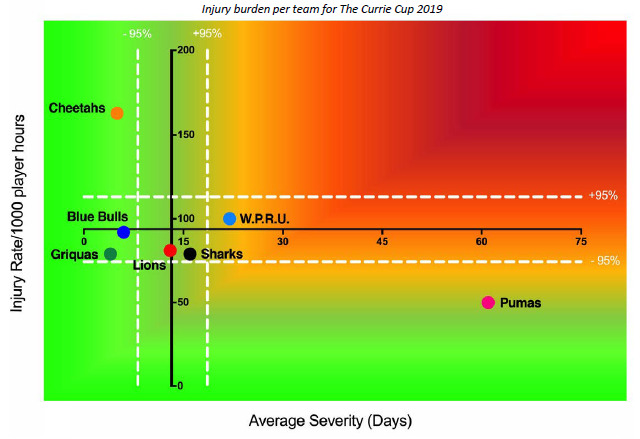
Injury rate plotted against the average severity of Time-Loss injuries for each participating team in The Currie Cup 2019. The Y-axis Average Injury Rate is expressed as the tournament average (±95% CI) and X-axis Average Severity is expressed as the average (±95% CI) of the individual injury severities in the tournament. Injury burden per team for The Currie Cup 2019

The Toyota Free State Cheetahs had the highest rate of Time-Loss injuries, but these were of low severity. Conversely the Phakisa Pumas had a low injury rate, but their injuries were of high severity (Figure 8). Teams who fall in the green zone, will generally not be impacted as much by their injury burden, regardless of whether their injury rate or average severity is relatively high. As soon as the combination of rate and severity moves into the orange and/or red zone, the impact on team performance and player availability becomes more problematic.

### Injury Type

Contusion/bruise injuries (22%, n = 21) were the most common Time-Loss injuries recorded in The Currie Cup 2019, with sprained ligament injuries comprising the second highest proportion (21%, n = 19). The median severity for contusion/bruise injuries was 3 days with 25% of injuries resulting in 2 or less days absent from training and matches, and 25% of injuries resulting in 5 or more days absent from training and matches (Table 5).

This is the first year in the six years surveyed that Contusion/bruise injuries have been the most common injury type. Central Nervous System injuries were the most common injury type in the 2018 tournament. Both the absolute number and average severity of Central Nervous System injuries decreased in the 2019 tournament (Table 6).

**Figure:**
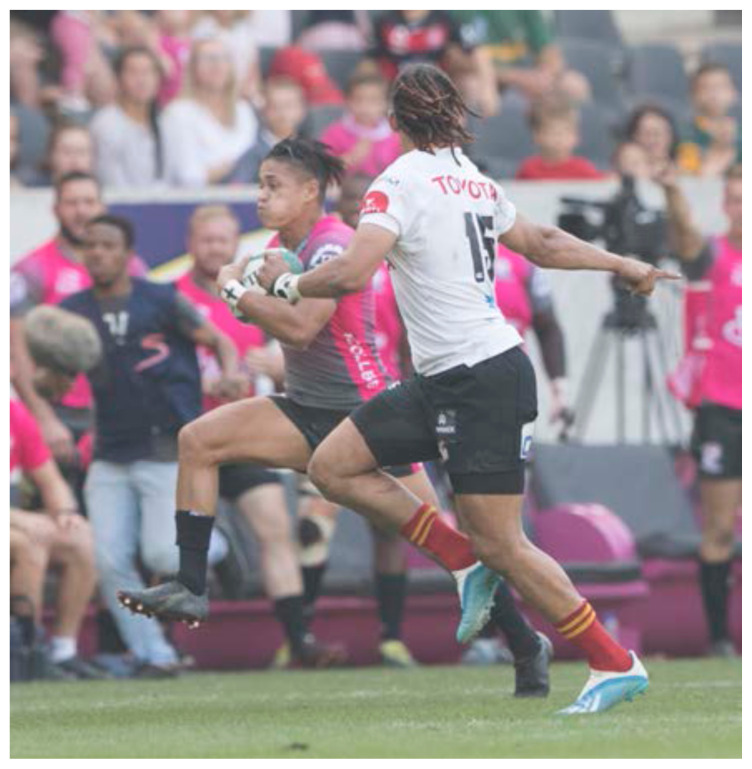


**Figure 9:**
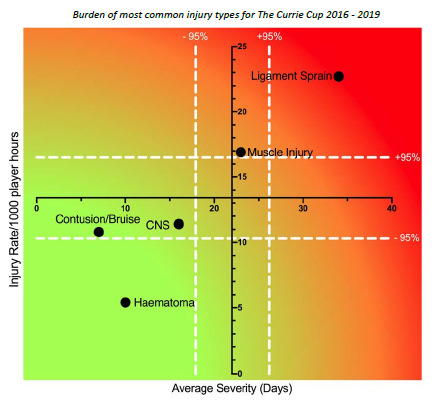
Injury rate plotted against the average severity of the most common Time-Loss injury types combined for 2016 to 2019. The Y-axis Average Injury Rate is expressed as the combined average for the plotted injuries (±95% CI) and X-axis Average Severity is expressed as the average of the individual injury severities for the plotted injuries (±95% CI). Burden of most common injury types for The Currie Cup 2016 – 2019

Combining the five most common injury types from 2016 – 2019 revealed that ligament sprain injuries are both the most frequently occurring injury type and have the highest average severity. While not as high as ligament injuries, muscle injuries also have a higher injury rate and average severity (Figure 9). Therefore, the effective burden, or the combination of injury rates and severities, of these two injury types still dominates, and therefore impacts teams more than the other injury types do.

For further comparison, The Currie Cup 2019 Time-Loss injury types were grouped in a similar way to the meta-analysis of international studies[1]. With these groupings, aligned to the meta-analysis, the most common Time-Loss injury types in The Currie Cup 2019 were joint (non-bone)/ligament injuries (comprised of dislocation/subluxation and sprain/ligament injuries).

The injury rate of 12 (5 to 18) injuries per 1000 player hours for the central nervous system during The Currie Cup 2019, was higher, although not significantly so, than the rate of “central/peripheral” system injuries of the meta-analysis, 8 (4 to 15) injuries per 1000 player hours[1].

The injury rate for muscle/tendon injuries (comprised of muscle rupture/strain/tear, tendon injury/rupture and tendinopathy injuries) was 19 (10 to 27) injuries per 1000 player hours. This was lower than the same type of injury grouping in the meta-analysis, which had a rate of 40 (21 to 76) injuries per 1000 player hours, albeit not significantly different[1]. The average severity for muscle/tendon injuries of 19 (3 to 36) days, in The Currie Cup 2019 was similar to the average severity of 15 (5 to 24) days reported in the meta-analysis[1].

In contrast, joint (non-bone)/ligament injuries (comprised of dislocation/subluxation and sprain/ligament injuries) were comparable: 20 (11 to 29) injuries per 1000 player hours in the present study, compared to the 24 (18 to 65) injuries per 1000 player hours in the meta-analysis[1]. The average severity of joint (non-bone)/ligament injuries in The Currie Cup 2019 was 15 (5 to 25) days, which is lower, but not significantly different, to the average severity for the same types of injuries reported at 29 (19 to 39) days in the meta-analysis[1].

**Figure:**
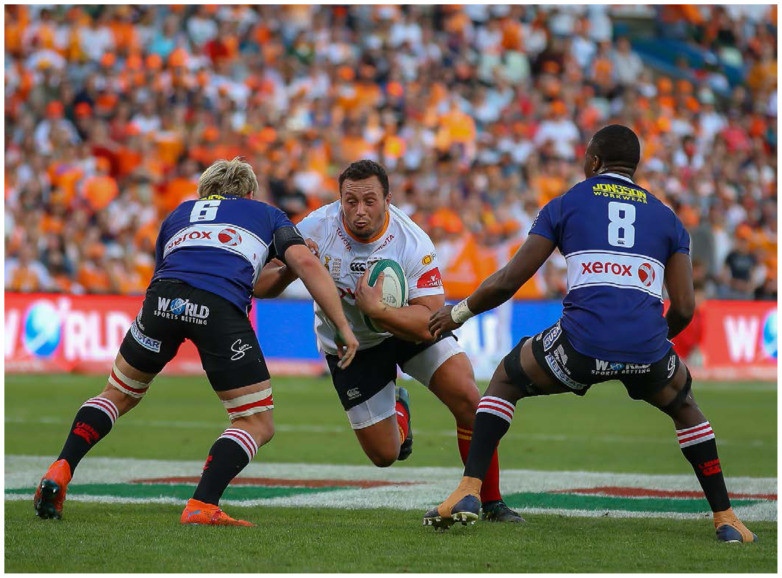


### Injury Diagnosis[7]

The most frequent OSICS classification diagnosis^7^ in The Currie Cup 2019 was HNCX Concussion. Concussions were the most frequently diagnosed injury in the 2014, 2016, 2017, 2018 and 2019 tournaments (Table 7).

### Concussions

**Figure 10:**
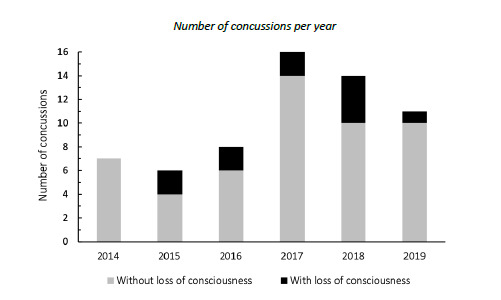
Number of concussions with and without loss of consciousness for The Currie Cup 2014 (n = 10), 2015 (n = 6), 2016 (n = 10), 2017 (n = 16), 2018 (n = 14) and 2019 (n = 11) tournaments. Number of concussions per year

Concussions contributed to 12% (n = 11) of all Time-Loss injuries for The Currie Cup 2019. Of these 11 concussions, only 2 (18%) were recorded with a loss of consciousness (Figure 10). The average severity of concussions reported in the 2019 tournament was 9 days (Range 5 – 20 days), which is lower than the average severity of 19 days reported for concussions in the England Professional Rugby Injury Surveillance Project 2017 – 2018 season report [3]. The current South African Rugby concussion regulations do not normally allow for adult players to return within less than 12 days of the concussive event. As this competition takes place at the professional level and is a World Rugby approved tournament, Advanced Care protocols are carried out by the medical practitioner that could potentially allow a player to return to play in less than 12 days.

Advanced care clinical settings are defined in the World Rugby and SARU’s Concussion Guideline documents:

- World Rugby Concussion Guideline document - https://playerwelfare.worldrugby.org/
- SARU’s Concussion Guideline documents (When can a player safely return to play following a concussion) www.boksmart.com/concussion

### Region of Injury

The most frequently injured body location of all Time-Loss injuries during The Currie Cup 2019 was the Head (20%, n = 19), followed by the Knee (11%, n = 11). Injuries to the head comprised on concussion (n = 13), bruising (n = 3) and lacerations (n = 5). The average burden of head injuries in 2019 was 128 days absent per 1000 player hours. This translates to an operational burden of 3 days lost per head injury per match over the entire season. The median severity of head injuries in 2019 was 6 days absent, with 25% of head injuries resulting in 2 or less days lost from training and matches and 25% of all head injuries resulting in 9 or more days lost from training and matches (Table 8).

When looking at the movement of the most common body locations of Time-Loss injuries for The Currie Cup 2014–2019 tournaments, the head and knee have remained the first and second most commonly injured body locations respectively from 2017–2019. While the absolute number and incidence of injuries to the head have increased slightly in 2019 from 2018, the average severity has dropped (Table 9).

**Figure:**
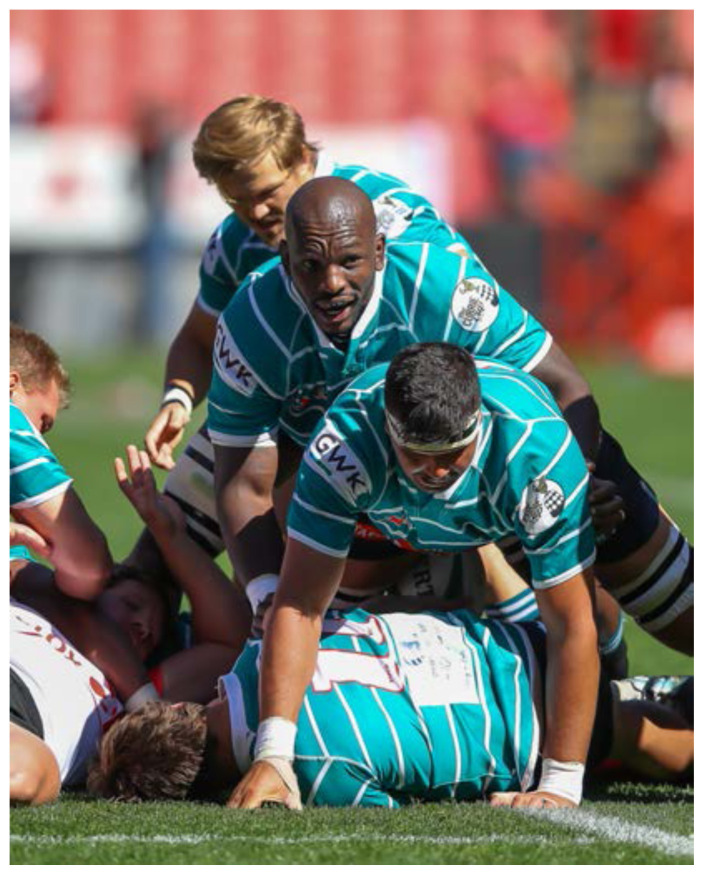


In The Currie Cup 2017 report the gradual increase in proportion of injuries to the shoulder was highlighted as a potential area of concern given the high average severity these injuries carry. In the 2018 tournament the shoulder remained the second most commonly injured body location. The proportion of shoulder injuries remained the same in 2018, but the average severity (38 days lost) was the lowest it had been since 2016. It is noteworthy that the shoulder does not feature in the top 5 most commonly injured body locations in 2019, with shoulder injuries making up 4% (n = 4) of all Time-Loss injuries. Despite the relatively low number of shoulder injuries, the average severity remains high at 52 days lost.

**Figure 11:**
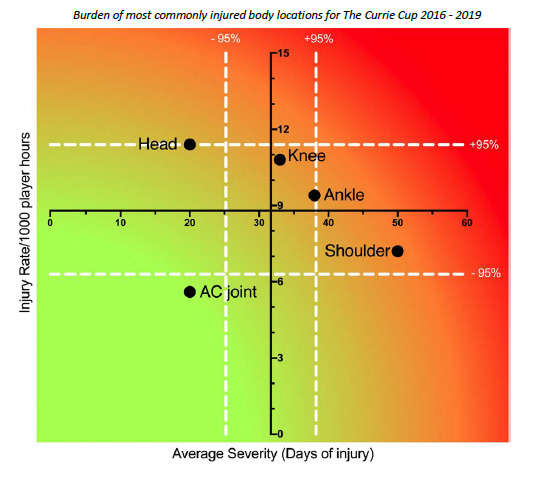
Injury rate plotted against the average severity of the most common body locations of Time-Loss injuries combined for 2016 to 2019. The Y-axis Average Injury Rate is expressed as the combined average for the plotted injuries (±95% CI) and X-axis Average Severity is expressed as the average of the individual injury severities for the plotted injuries (±95% CI). Burden of most commonly injured body locations for The Currie Cup 2016 – 2019

Combining the most commonly injured body locations from 2016–2019 revealed that injuries to the shoulder have a moderate injury rate but are of a high average severity. Conversely, injuries to the head have a high injury rate, but comparatively do not have a high average severity (Figure 11). Knee and ankle injuries due to their higher burden of injury, still require notable attention.

**Figure 12:**
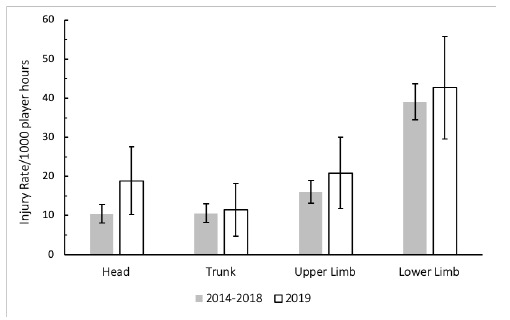
The Time-Loss injury rates of the four most common grouped body locations (injuries/1000 player hours) for The Currie Cup 2019 in comparison to the averaged 2014–2018 injury rates.

When anatomical body locations were grouped for comparison with the data from the meta-analysis[1], the lower limb recorded the highest injury rate for The Currie Cup 2019 (Figure 12). In 2019, the injury rate to the lower limb of 43 (30 to 56) injuries per 1000 player hours was similar to that of the meta-analysis[1] at 47 (28 to 46) injuries per 1000 player hours. The injury rates for all grouped body locations were higher in The Currie Cup 2019 than the average 2014–2018 injury rates, although these were not significant. The upper limb recorded the highest average severity of 26 days per injury, followed by the trunk at 11 days per injury.

**Figure:**
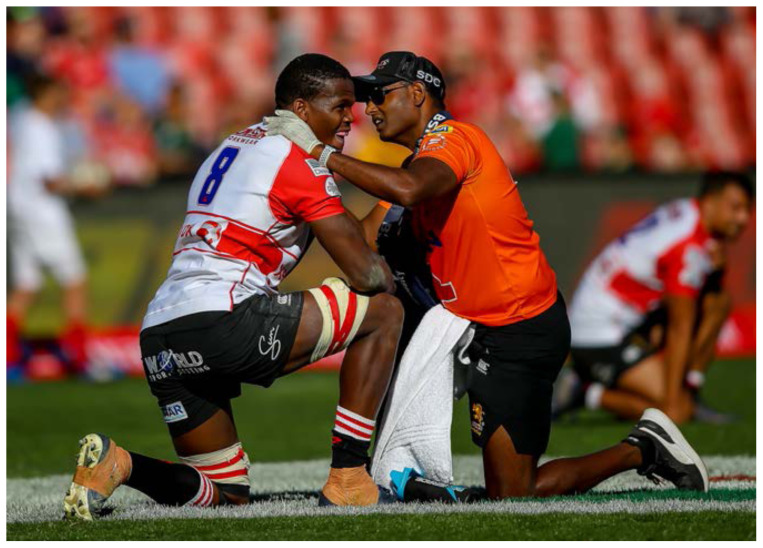


### Injury Event

In The Currie Cup 2019, *tackling* (i.e. performing the tackle) and *being tackled* (i.e. to the ball carrier) accounted equally for the highest proportion (22%, n = 20 each) of injury events, with an injury rate of 21 (12 to 30) injuries per 1000 player hours for each event. This injury rate is comparable to the meta-analysis[1] injury rate for *tackling* of 19 (12 to 29) injuries per 1000 player hours, but is higher than the injury rate for *being tackled* of 12 (5 to 19) injuries per 1000 player hours.

**Figure 13:**
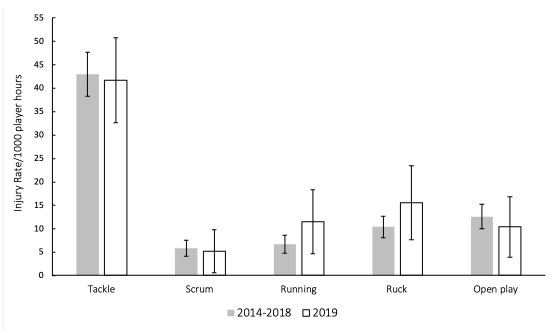
Injury rate (± 95% CI) of the most common injury causing events for The Currie Cup 2019 in comparison to the averaged 2014–2018 tournament injury rates.

In The Currie Cup 2014 – 2019 tournaments, the tackle phase accounted for the highest proportion of all injury events (Figure 13). There were no significant differences in the injury rates of the most common injury causing events in The Currie Cup 2019 in comparison to the average 2014–2018 injury rates (Figure 13), albeit that injuries from running and the ruck appeared to increase slightly.

For each year of The Currie Cup tournament, *‘tackling front-on’ has* accounted for the highest proportion of tackling related injuries; 2014 (44%, n = 23), 2015 (33%, n = 13), 2016 (30%, n = 17), 2017 (30%, n = 13), 2018 (35%, n = 8) and 2019 (25%, n = 10).

### Playing Position of Injured Players

In The Currie Cup 2019, forwards had a Time-Loss injury rate of 65 (55 to 74) injuries per 1000 player hours, while for the backs it was 28 (18 to 39) injuries per 1000 player hours. When comparing these rates with the meta-analysis[1], the 2019 rates for both forwards and backs were lower than those reported in the meta-analysis.

**Figure 14:**
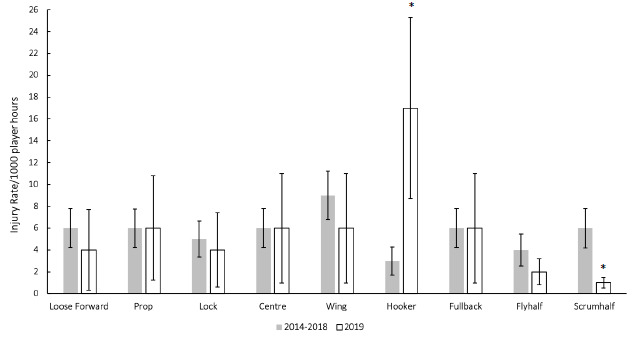
Injury rates (± 95% CI) normalised per playing position for The Currie Cup 2019 in comparison to the averaged 2014–2018 tournament injury rates. *Indicates significant difference to the 2014–2018 injury rate.

When divided into specific positional groupings within forwards and backs, the number of injuries were normalised relative to the number of players on the field per position per team (e.g. 2 Props = total injuries divided by 2; 3 Loose Forwards = total injuries divided by 3). In The Currie Cup 2019 there were significantly more injuries sustained by Hookers and significantly less injuries sustained by Scrumhalves than the average 2014–2018 injury rate (Figure 14). However, with the relatively low numbers, it is difficult to draw any firm conclusions.

### Protective Gear and Injury

The majority (40%) of all players who sustained a Time-Loss injury in The Currie Cup 2019 were wearing a mouth guard. Of the 11 concussions, four of them occurred with the player wearing a mouth guard. The number of players who participated in the match wearing a mouth guard and those who did not wear a mouth guard is unknown. Therefore, one cannot draw any firm conclusions about the associations between wearing a mouth guard and injury. In 2019, doctors or medical staff were asked to specify if strapping was worn on the injured body location. It was specified that strapping was worn on the injured body location for 12 injuries (13%). Of these 12 injuries, four were ankle sprains. For 36% of injuries recorded however, information pertaining to protective gear worn was not captured. We have highlighted the concern about the medical practitioners not entering protective gear data and will discuss this with them to identify a solution to improve compliance in the future.

### Venue

**Figure 15:**
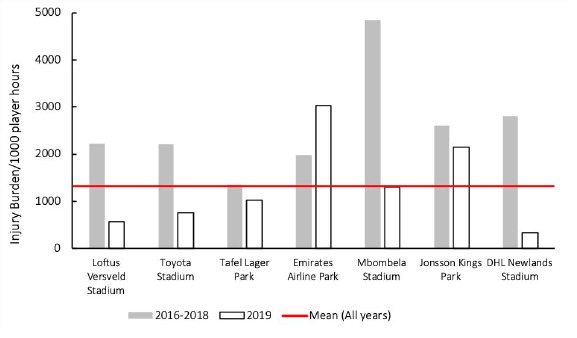
Injury burden/1000 player hours of Time-Loss injuries at the seven utilised stadia in The Currie Cup 2019 in comparison to their averaged 2016–2018 injury burden.

Matches were played at seven stadia during the tournament. For The Currie Cup 2019, Emirates Airline Park, Johannesburg and Jonsson Kings Park, Durban had an injury burden higher than the 2016–2019 average tournament injury burden, however the injury burden at Jonsson Kings Park was lower in 2019 than their 2016–2018 averaged burden (Figure 15). Injury burden at Loftus Versfeld, Toyota Stadium, Mbombela Stadium and Newlands Stadium was also lower in 2019 than their 2016–2018 averaged injury burden.

**Figure 16:**
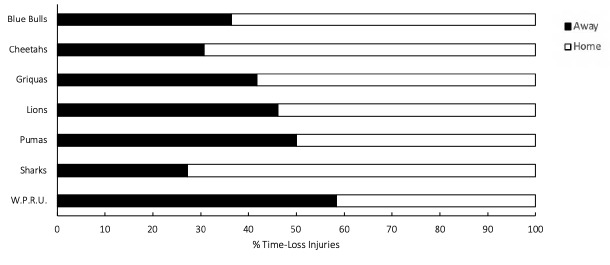
Proportion of injuries sustained playing at home and away venues for The Currie Cup 2019 Premiership Division Competition (n = 90).

Across all teams, and although not significant, playing at home (57 [42 to 71] injuries per 1000 player hours) had a higher injury rate than playing away (36 [24 to 49] injuries per 1000 player hours) in The Currie Cup 2019 tournament. The Phakisa Pumas showed equal proportions of injuries playing both away and at home. DHL Western Province sustained more injuries playing away than at home, while all other teams sustained more injuries playing at home (Figure 16).

**Figure:**
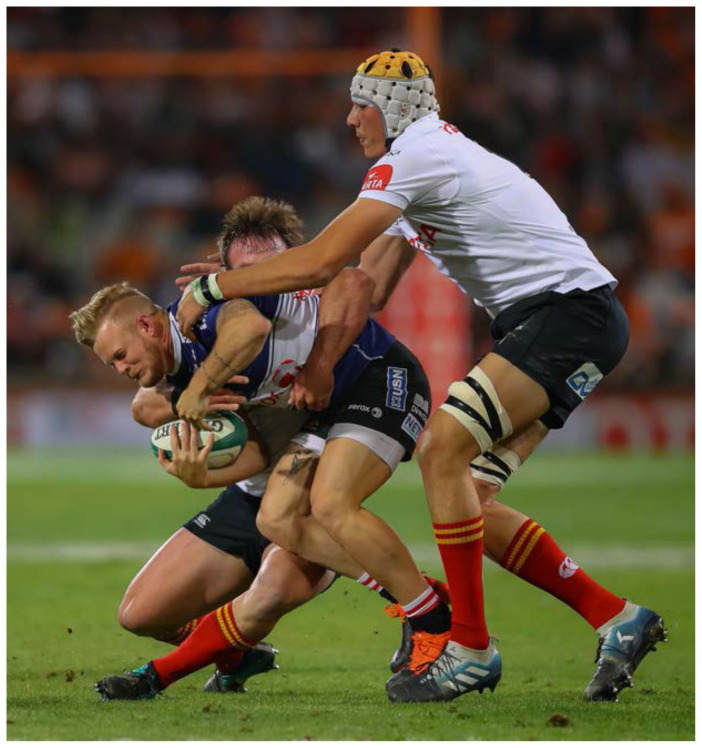


## Summary

The injury rate of Time-Loss injuries for The Currie Cup 2019 was 94 (74 to 113) injuries per 1000 player hours, which is higher than the rate of the meta-analysis of international studies, 81 (63 to 105) injuries per 1000 player hours[1], and equates to 1.9 injuries per team per match. The average severity of Time-Loss injuries was 13 days lost, and the median severity was 4 days (IQR 3 to 10). The mean and 95% CI Time-Loss injury rates for all teams combined for the 2014 – 2019 tournaments [83 (77 to 89) injuries per 1000 player hours] is very similar to that of the meta-analysis [81 (63 to 105) injuries per 1000 player hours].

Most of the data in The Currie Cup 2019 Premiership Division Competition were similar to the data shown in the meta-analysis [1], illustrating a similarity with international trends. The injury rate of recurrent Time-Loss injuries in The Currie Cup 2019 Premiership Division Competition was significantly lower than that of the meta-analysis. An interesting point to reflect upon is the following. Of the 90 Time-loss injuries in 2019, 13 players (8% of the player pool) contributed to 28 (31%) of all the 2019 Tournament’s injuries. The potential impact of this finding needs further interrogation and introspection within the Unions to ascertain why certain players are more prone to injury and/or re-injury than others.

Despite having the highest Time-Loss injury rate Toyota Free State Cheetahs had the second lowest average injury severity of 5 days. The median severity of injuries sustained in Toyota Free State Cheetahs was 3 days with 25% of their injuries losing 2 days or less and only 25% of their injuries losing 4 days or more of rugby training and match play. This means that they had a high number of injuries but that the average severity of their injuries was low resulting in them not losing many training and match days for each injury. In contrast, the Phakisa Pumas had the lowest Time-Loss injury rate, but the highest average injury severity of 61 days. They had a median injury severity of 27 days with 25% of their injuries losing 14 days or less of rugby and 25% of their injuries losing 151 days or more. This means that even though the Phakisa Pumas had a low number of injuries, many training and match days were lost due to those injuries.

It is important to note that the return-to-play date of an injured player will, to an extent, be influenced by the rehabilitation approach taken by the team. Teams with a more conservative approach to the rehabilitation of injuries will reflect an increased total injury severity. The results of this study highlight the importance of collecting severity data, and not simply injury rates on their own, as although teams may have a low injury rate, injuries of a high severity still represent a sizable burden to the team resulting in a large number of training and match days lost due to injury. What is interesting about this finding, is that although the Toyota Free State Cheetahs had the highest injury rate, they won the tournament and the Phakisa Pumas, who had the lowest injury rate finished at the bottom of the log. This injury trend is the opposite to what has been seen in previous years and in international studies [2], [8]. In previous years the teams who ranked in 1^st^ or 2^nd^ position had significantly lower injury rates than those who ranked in last position [2].

When combining all injuries from 2014 – 2018; 58% of all *moderate* and *severe* injuries occurred to the head, knee, ankle, shoulder and AC joint areas. We have recently started a video analysis project to further analyse and describe the mechanisms contributing to these injuries. As mentioned in the 2018 report, this will remain a focus area over the next few years.

Injury surveillance is the critical first step in the development of injury prevention strategies for a surveyed group. After six years of injury surveillance in The Currie Cup competition these data now provide a well powered dataset and provide a strong evidence-base for developing targeted injury prevention strategies. SARU is encouraged to continually collaborate with all key role players and identify those key areas that would benefit from practical solution injury prevention interventions.

**Figure:**
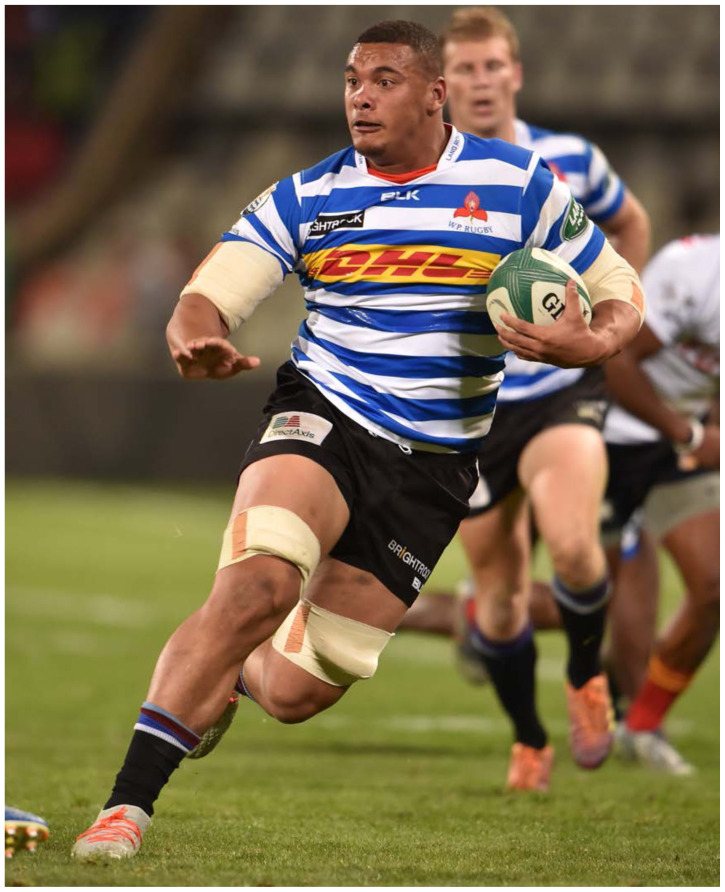


**Figure:**
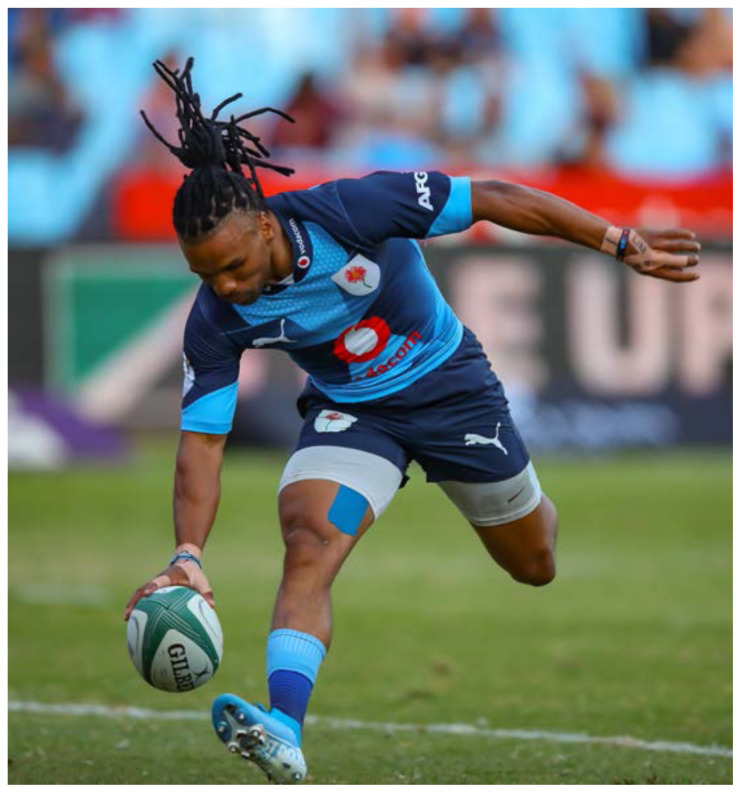


## Figures and Tables

**Figure 1a:**
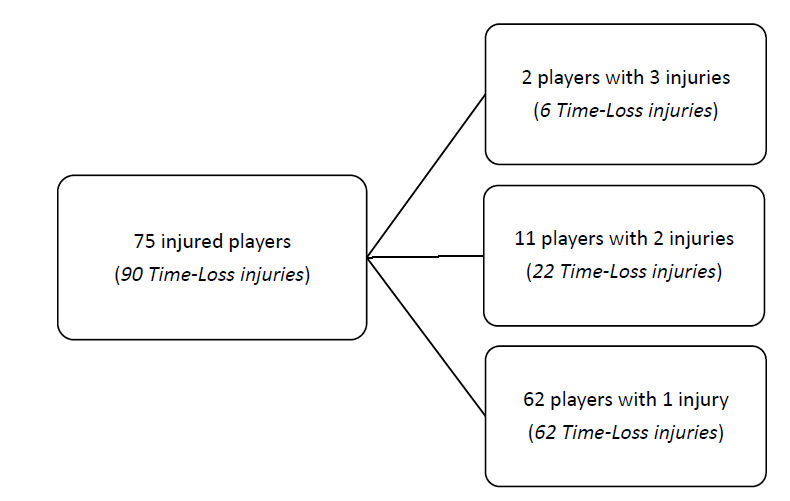
The number of players who experienced Time-Loss injuries during The Currie Cup 2019.

**Figure 1b:**
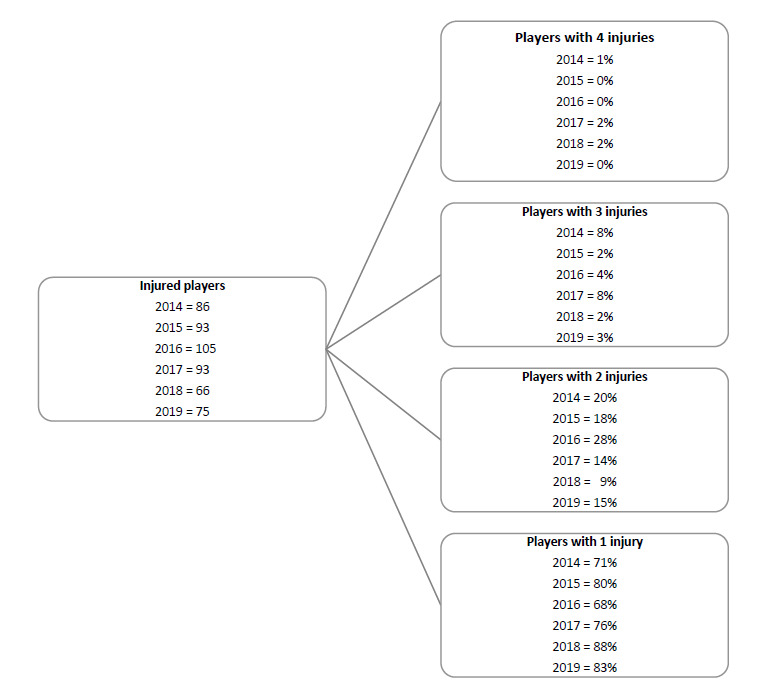
Proportion of players who experienced Time-Loss injuries in The Currie Cup tournaments from 2014–2019.

**Figure 2a:**
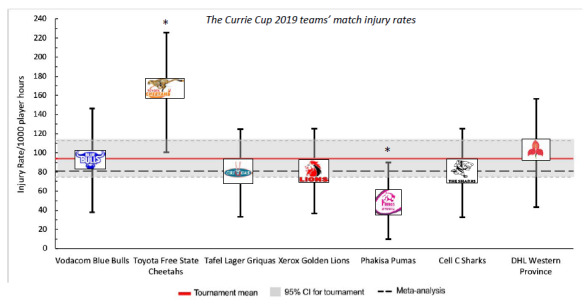
Match Time-Loss injury rates of the seven teams participating in The Currie Cup 2019, including the mean and 95% confidence intervals (CI) for all teams combined. Time-Loss injuries; n = 90. Asterisk (*) indicates that a team’s injury rate is significantly different to another team in the tournament for that year.

**Figure 2b:**
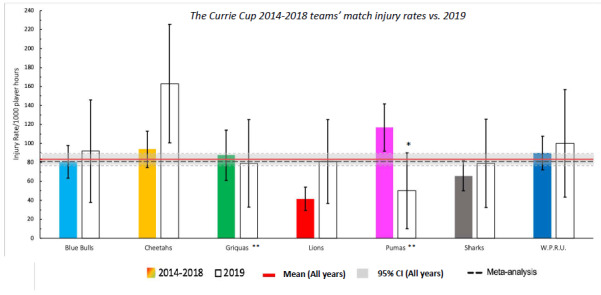
Injury Rate/1000 player hours for Time-Loss injuries experienced by each team in The Currie Cup 2019 in comparison to their 2014–2018 averaged injury rate. (**) Average injury rates for Pumas 2015 – 2018 and Griquas for 2015, 2016 and 2018. Asterisk (*) indicates that a team’s 2019 injury rate is significantly different to their 2014–2018 averaged injury rate.

**Figure 4a:**
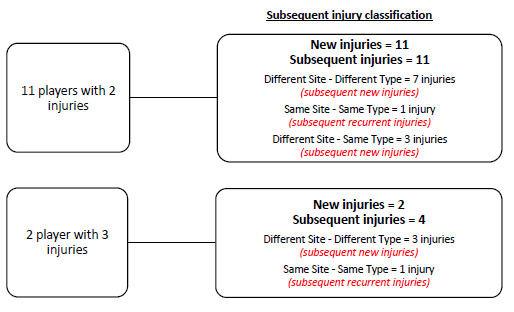
Classification of subsequent Time-Loss injuries sustained during The Currie Cup 2019 (13 players sustained more than one injury; the first injury for each player was recorded as a new injury and the following as either subsequent new or subsequent recurrent injuries. New injuries n = 13; subsequent new injuries n = 13; subsequent recurrent injuries n = 2).

**Figure 4b:**
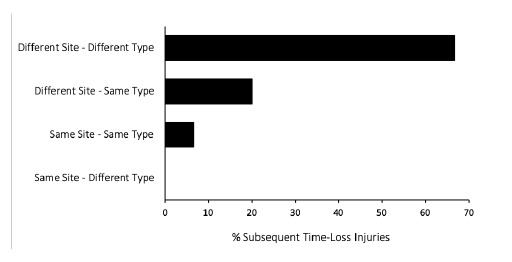
Classification of subsequent Time-Loss injuries for The Currie Cup 2019. Data expressed as a % of subsequent Time-Loss injuries.

**Table 1:** Injury rate per team, risk per player and risk per team for Time-Loss injuries in The Currie Cup 2019.

Team	Injury Rate (95% CI)	Team Injuries/Match	Player match hours/Team injury	Chances of injury per player per match (%)	Number of matches per player before an injury can be expected
Vodacom Blue Bulls	92 (37 to 146)	1.8	10.9	12	8.2
Toyota Free State Cheetahs	163 (100 to 225)	3.3	6.1	22	4.6
Xerox Golden Lions	81 (37 to 125)	1.6	12.3	11	9.3
Phakisa Pumas	50 (10 to 90)	1.0	20.0	7	15.0
Cell C Sharks	79 (32 to 125)	1.6	12.7	11	9.5
DHL Western Province	100 (43 to 157)	2.0	10.0	13	7.5
Tafel Lager Griquas	79 (32 to 125)	1.6	12.7	11	9.5

OVERALL	94 (74 to 113)	1.9	10.6	13	8.0

**Table 2:** The Currie Cup 2019 injuries grouped according to the IOC recommended categories of Tissue and Pathology types for injuries

Tissue	Injuries	Incidence	Median time loss	Burden

*Pathology*	n	Injuries per 1000 hours (95% CI)	Days (95% CI)	Time loss days per 1000 hours (95% CI)
**Muscle/Tendon**	39	40.6 (28 to 53)	3 (5 to 23)	572 (547 to 645)
* Muscle injury*	16	16.7 (9 to 25)	4 (2 to 26)	231 (210 to 272)
* Muscle contusion*	20	20.8 (12 to 30)	3 (2 to 8)	100 (84 to 125)
* Tendinopathy*	3	3.1 (0 to 7)	61 (1 to 153)	241 (219 to 282)
**Nervous**	11	11.5 (5 to 18)	9 (7 to 12)	105 (89 to 131)
* Brain/Spinal cord injury*	11	11.5 (5 to 18)	9 (7 to 12)	105 (89 to 131)
**Bone**	7	7.3 (2 to 13)	9 (0 to 75)	266 (243 to 310)
* Fracture*	4	4.2 (0 to 8)	48 (1 to 120)	252 (230 to 295)
* Bone stress injury*	3	3.1 (0 to 7)	2 (0 to 9)	14 (7 to 22)
**Cartilage/Synovium/Bursa**	4	4.2 (0 to 8)	4 (3 to 5)	17 (9 to 26)
* Cartilage injury*	3	3.1 (0 to 7)	3 (2 to 6)	13 (6 to 20)
* Synovitis/Capsulitis*	1	1.0 (0 to 3)	4	4 (0 to 9)
**Ligament/Joint capsule**	20	20.8 (12 to 30)	9 (7 to 18)	263 (240 to 307)
* Joint sprain*	1	1.0 (0 to 7)	1	3 (0 to 7)
* Ligament sprain*	19	19.8 (11 to 29)	9 (7 to 18)	263 (237 to 303)
**Superficial tissue/skin**	9	9.4 (3 to 16)	2 (2 to 4)	26 (17 to 38)
* Contusion (superficial)*	3	3.1 (0 to 7)	2 (2 to 2)	6 (1 to 12)
* Laceration*	6	6.3 (1 to 11)	3 (1 to 5)	20 (12 to 30)

Where n = 1, median time-loss reflects the total time-loss days.

**Table 3:** Injury pattern and burden of specific match injuries in The Currie Cup 2019.

Region Type *Diagnosis*	Injuries	Incidence	Median time-loss	Burden

	n	Injuries per 1000 hours (95%CI)	Days (95% CI)	Time loss days per 1000 hours (95%CI)
Head	19	19.8 (11 to 29)	6 (4 to 8)	127 (109 to 155)
* Concussion*	11	11.5 (5 to 18)	9 (7 to 12)	105 (89 to 131)
Head bruising	3	3.1 (0 to 7)	2 (2 to 2)	6 (1 to 12)
Head laceration	5	5.2 (1 to 10)	2 (1 to 5)	16 (8 to 24)
**Neck**	1	1.0 (0 to 3)	61	64 (50 to 82)
Neck muscle spasm	1	1.0 (0 to 3)	61	64 (50 to 82)
**Shoulder**	8	8.3 (3 to 14)	7 (2 to 20)	92 (76 to 115)
* Acute dislocation*	1	1.0 (0 to 3)	12	13 (6 to 20)
Haematoma	3	3.1 (0 to 7)	3 (2 to 3)	8 (3 to 15)
Muscle strain	1	1.0 (0 to 3)	42	44 (32 to 59)
* Acromioclavicular jt sprain*	3	3.1 (0 to 7)	9 (5 to 12)	27 (18 to 39)
**Upper arm**	4	4.2 (0 to 8)	3 (0 to 105)	165 (145 to 198)
Muscle contusion	3	3.1 (0 to 7)	2 (2 to 3)	7 (2 to 13)
Tendon injury	1	1.0 (0 to 3)	151	157 (138 to 189)
**Elbow**	2	2.1 (0 to 5)	51 (0 to 123)	105 (89 to 131)
Muscle strain	2	2.1 (0 to 5)	51 (0 to 123)	105 (89 to 131)
**Forearm**	2	2.1 (0 to 5)	74 (0 to 188)	153 (134 to 185)
Fracture	1	1.0 (0 to 3)	145	151 (132 to 182)
Muscle contusion	1	1.0 (0 to 3)	2	2 (0 to 5)
**Wrist and hand**	4	4.2 (0 to 8)	4 (3 to 4)	16 (8 to 24)
Dislocation	1	1.0 (0 to 3)	4	4 (0 to 9)
Laceration	1	1.0 (0 to 3)	4	4 (0 to 9)
Muscle strain	2	2.1 (0 to 5)	4 (3 to 4)	7 (2 to 13)
**Chest**	6	6.3 (1 to 11)	3 (2 to 7)	28 (18 to 40)
Fracture	1	1.0 (0 to 3)	2	2 (0 to 5)
Stress fracture	3	3.1 (0 to 7)	2 (0 to 8)	14 (7 to 22)
* Sternoclavicular joint sprain*	1	1.0 (0 to 3)	3	3 (0 to 7)
Muscle contusion	1	1.0 (0 to 3)	9	9 (4 to 16)
**Lumbar spine**	1	1.0 (0 to 3)	19	20 (12 to 30)
Muscle strain	1	1.0 (0 to 3)	19	20 (12 to 30)
**Hip/Groin**	2	2.1 (0 to 5)	3 (2 to 3)	5 (1 to 10)
Muscle strain	2	2.1 (0 to 5)	3 (2 to 3)	5 (1 to 10)
* Groin muscle strain*	1	1.0 (0 to 3)	2	2 (0 to 5)
* Hip flexor muscle strain*	1	1.0 (0 to 3)	3	3 (0 to 7)
**Thigh**	9	9.4 (3 to 16)	4 (2 to 8)	50 (38 to 67)
* Hamstring strain*	4	4.2 (0 to 8)	5 (1 to 12)	28 (18 to 40)
Haematoma	5	5.2 (1 to 10)	3 (2 to 7)	22 (13 to 32)
**Knee**	11	11.5 (5 to 18)	6 (3 to 20)	133 (115 to 162)
Knee cartilage injury	4	4.2 (0 to 8)	4 (3 to 5)	17 (9 to 26)
* Meniscal cartilage*	3	3.1 (0 to 7)	3 (2 to 6)	13 (6 to 20)
* Synovitis*	1	1.0 (0 to 3)	4	4 (0 to 9)
Knee ligament injury	5	5.2 (1 to 10)	16 (6 to 36)	111 (95 to 138)
* MCL injury*	3	3.1 (0 to 7)	9 (7 to 15)	35 (25 to 49)
* Not specified*	2	2.1 (0 to 5)	37 (8 to 65)	76 (61 to 97)
Muscle contusion	2	2.1 (0 to 5)	3 (2 to 3)	5 (1 to 10)
**Lower leg**	10	10.4 (4 to 17)	4 (3 to 24)	142 (123 to 172)
Fracture	1	1.0 (0 to 3)	57	59 (46 to 78)
Muscle contusion	4	4.2 (0 to 8)	3 (0 to 22)	41 (29 to 55)
Muscle injury	5	5.2 (1 to 10)	4 (3 to 13)	42 (30 to 57)
* Calf cramping*	2	2.1 (0 to 5)	4 (3 to 4)	7 (2 to 13)
* Calf muscle spasm*	1	1.0 (0 to 3)	4	4 (0 to 9)
* Gastrocnemius strain*	2	2.1 (0 to 5)	15 (12 to 17)	30 (20 to 43)
**Ankle**	10	10.4 (4 to 17)	3 (2 to 15)	89 (73 to 111)
Ankle sprain	9	9.4 (3 to 16)	3 (2 to 16)	83 (68 to 105)
* Deltoid ligament sprain*	3	3.1 (0 to 7)	3 (3 to 3)	9 (4 to 16)
* Lateral ligament sprain*	1	1.0 (0 to 3)	3	3 (0 to 7)
* Syndesmosis sprain*	5	5.2 (1 to 10)	9 (2 to 25)	71 (57 to 91)
Muscle contusion	1	1.0 (0 to 3)	5	5 (1 to 10)
**Foot**	1	1.0 (0 to 3)	38	40 (28 to 54)
* Metatarsal fracture*	1	1.0 (0 to 3)	38	40 (28 to 54)

Where n = 1, median time-loss reflects the total time-loss days. MCL, medial collateral ligament.

**Table 4:** Severity (days), Injury Burden (days absent/1000 player hours) and Operational Burden (days absent/injury/match) of Time-Loss injuries for each participating team in The Currie Cup 2019.

Team	Team Injuries/match	Team matches/injury	Total Severity	Average Severity	Injury Burden	Operational Injury Burden	Median Severity (IQR)
Vodacom Blue Bulls	1.8	0.5	67	6	560	11	9 (2 to 9)
Toyota Free State Cheetahs	3.3	0.3	121	5	759	15	3 (2 to 4)
Xerox Golden Lions	1.6	0.6	165	13	1028	21	12 (6 to 16)
Phakisa Pumas	1.0	1.0	363	61	3025	61	27 (14 to 151)
Cell C Sharks	1.6	0.6	181	16	1300	26	5 (4 to 19)
DHL Western Province	2.0	0.5	258	22	2150	43	8 (4 to 23)
Tafel Lager Griquas	1.6	0.6	43	4	309	6	3 (3 to 3)

OVERALL	1.9	0.5	1198	13	1251	25	4 (3 to 10)

**Table 5:** Injury rate, Severity and Burden of the most common injury types in The Currie Cup 2019.

Injury Type	Injury Rate (95% CI)	Total Severity	Average Severity	Average Burden	Operational Injury burden	Median (IQR)
Contusion/Bruise	22 (13 to 31)	160	8	168	3	3 (2 to 5)
Sprained Ligament	20 (11 to 29)	249	13	262	5	9 (3 to 16)
Muscle (Rupture/Strain/Tear)	16 (8 to 24)	124	8	132	6	4 (3 to 13)
Central Nervous System	12 (5 to 18)	101	9	110	2	9 (6 to 10)
Fractures	7 (2 to 13)	255	36	255	5	9 (2 to 48)
Lacerations	6 (1 to 11)	19	3	19	0.4	3 (2 to 4)

**Table 6:** The movement of the most common types of Time-Loss Injuries from 2014 – 2019. Data expressed as a %, absolute number and incidence of Time-Loss injuries for 2014 (n = 120), 2015 (n = 114), 2016 (n = 142), 2017 (n = 126), 2018 (n = 77), 2019 (n = 90) and the average severity for 2016–2019 is expressed in days.

			%	Number	Incidence	
2014		Sprain Ligament	28	33	25 (17–34)	
		Muscle (Rupture/Strain/Tear)	18	21	16 (9–23)	
		Haematoma	11	13	10 (5–15)	
		Contusion/Bruise	9	11	8 (3–13)	
		Dislocation/Subluxation	8	10	8 (3–12)	
		Central Nervous System	8	10	8 (3–12)	
2015		Sprain Ligament	34	39	23 (16–30)	
		Muscle (Rupture/Strain/Tear)	18	21	12 (7–17)	
		Contusion/Bruise	16	18	11 (6–15)	
		Central Nervous System	7	8	5 (1–8)	
		Haematoma	5	6	4 (1–6)	
						Average Severity
2016		Sprain Ligament	30	43	28 (19–36)	34
		Muscle (Rupture/Strain/Tear)	20	29	19 (12–25)	23
		Contusion/Bruise	11	16	10 (5–15)	6
		Central Nervous System	8	11	7 (3–11)	23
		Haematoma	6	9	6 (2–10)	18
2017		Sprain Ligament	33	42	27 (19–35)	43
		Muscle (Rupture/Strain/Tear)	23	29	19 (12–25)	28
		Central Nervous System	13	17	11 (6–16)	14
		Haematoma	9	11	7 (3–11)	8
		Contusion/Bruise	6	8	5 (2–9)	13
2018		Central Nervous System	25	19	20 (11–29)	21
		Sprain Ligament	18	14	15 (7–23)	39
		Muscle (Rupture/Strain/Tear)	16	12	13 (6–20)	25
		Haematoma	8	6	6 (1–12)	6
		Contusion/Bruise	6	5	5 (1–10)	4
2019		Contusion/Bruise	23	21	22 (13–31)	8
		Sprain Ligament	21	19	20 (11–29)	13
		Muscle (Rupture/Strain/Tear)	17	15	16 (8–24)	8
		Central Nervous System	12	11	12 (5–18)	9
		Fracture	8	7	7 (2–13)	36
		Laceration	7	6	6 (1–11)	3

**Table 7:** The movement of the most common OSICS classification diagnoses[7] of Time-Loss Injuries from 2014 – 2019. Data expressed as a %, absolute number and incidence of total Time-Loss injuries for 2014 (n = 120), 2015 (n = 114), 2016 (n = 142), 2017 (n = 126), 2018 (n = 77) and 2019 (n = 90); the average severity for 2016–2019 is expressed in days.

			%	Number	Incidence	
2014		HN1 Concussion	7	8	6 (2–10)	
		THV Quadriceps haematoma	6	7	5 (1–9)	
		KL3 Knee medial collateral ligstr/tear/rupture	6	7	5 (1–9)	
2015		SJ2 Acromioclavicular jt sprain	7	8	5 (1–8)	
		KL3 Knee medial collateral ligstr/tear/rupture	7	8	5 (1–8)	
		THV Quadriceps haematoma	5	6	3 (1–6)	
		HN1 Concussion	5	6	3 (1–6)	
						Average Severity
2016		HN1 Concussion	7	10	6 (2–10)	14
		KL3 Knee medial collateral ligstr/tear/rupture	6	9	6 (2–10)	23
		TM1 Hamstring strain/tear	6	8	5 (2–9)	11
2017		HNCX Concussion	13	16	10 (5–15)	15
		SJAX Acromioclavicular jt sprain	10	12	8 (3–12)	25
2018		HNCX Concussion	18	14	15 (7–23)	14
		TMQX Quadricep strain	5	4	4 (0–8)	18
2019		HNCX Concussion	12	11	12 (5–18)	9
		AJSX Ankle syndesmosis sprain	5	5	5 (1–10)	14

**Table 8:** Injury rate, Severity and Burden of the most common injury types in The Currie Cup 2019.

Injury Type	Injury Rate (95% CI)	Total Severity	Average Severity	Average Burden	Operational Injury Burden	Median (IQR)
Head	20 (11 to 29)	122	6	128	3	6 (2 to 9)
Knee	12 (5 to 18)	128	12	140	3	6 (6 to 13)
Ankle	10 (4 to 17)	85	9	85	2	3 (3 to 9)
Lower limb posterior	6 (1 to 11)	20	3	20	0.4	3 (3 to 4)
Posterior thigh	6 (1 to 11)	56	9	56	1	9 (4 to 16)

**Table 9:** The movement of the most common body locations of Time-Loss Injuries from 2014 – 2019. Data expressed as a %, absolute number and incidence of total Time-Loss injuries for 2014 (n = 120), 2015 (n = 114), 2016 (n = 142), 2017 (n = 126), 2018 (n = 77), 2019 (n = 90) and the average severity for 2016–2019 expressed in days.

			%	Number	Incidence	
2014		Knee	14	17	13 (7–19)	
		Ankle	10	12	9 (4–14)	
		Head	9	11	8 (3–13)	
		Posterior Thigh	7	8	6 (2–10)	
		Anterior Thigh	7	8	6 (2–10)	
		A/C Joint	7	8	6 (2–10)	
2015		Knee	18	20	12 (7–17)	
		Ankle	9	10	6 (2–9)	
		A/C Joint	8	9	5 (2–9)	
		Head	7	8	5 (1–8)	
						Average Severity
2016		Knee	14	20	13 (7–18)	49
		Ankle	13	18	12 (6–17)	51
		Head	9	13	8 (4–13)	11
		Shoulder	8	12	8 (3–12)	41
2017		Head	13	16	10 (5–15)	15
		Knee	11	14	9 (4–14)	63
		Shoulder	10	12	8 (3–12)	67
		Ankle	10	12	8 (3–12)	87
		A/C Joint	10	12	8 (3–12)	25
2018		Head	18	14	15 (7–23)	18
		Knee	10	8	9 (3–14)	44
		Shoulder	10	8	9 (3–14)	38
		Ankle	9	7	7 (2–13)	65
		Anterior thigh	8	6	6 (1–12)	6
2019		Head	21	19	20 (11 – 29)	6
		Knee	12	11	12 (5 – 18)	12
		Ankle	11	10	10 (4 – 17)	9
		Lower limb posterior	7	6	6 (1 – 11)	3
		Posterior thigh	7	6	6 (1 – 11)	9

## Footnotes

7 Orchard, J. *Orchard Sorts Injury Classification System (OSICS)* 2019; Available from: https://www.johnorchard.com/osics-downloads.html.
